# The Delivery and Quality of Sexually Transmitted Infections Treatment by Private General Practitioners in Windhoek Namibia

**DOI:** 10.5539/gjhs.v4n5p156

**Published:** 2012-08-13

**Authors:** Scholastika N Iipinge, Louise Pretorius

**Affiliations:** 1 School of Nursing and Public Health, University of Namibia, Windhoek, Namibia

**Keywords:** private general practitioner (PGP), STI treatment, treatment guidelines, Windhoek, Namibia

## Abstract

**Introduction:**

The main objective for this study was to investigate the quality of Sexually Transmitted Infections (STI) treatment and control by the private sector in Namibia.

**Method:**

This was a cross-sectional study employing quantitative methodology using different methods of data collection. A self-administered questionnaire exploring General Practitioners (GPs) perceptions of factors that influence the way they manage Sexually Transmitted Infections (STI) which was then concluded with the face to face interviews and the checklist that was used while doing observations in the consulting rooms

**Results:**

A total of 50 private general practitioners in the area of Windhoek were interviewed, 48 self-administered questionnaires plus all checklists were received back from the private general practitioners.

None of the private general practitioners interviewed had specific training in the syndromic management of the STIs. The 86% of all patients were seen by these private general practitioners on a medical aid, while 14 % pay cash for service provided.

With regard to Urethral Discharge, an average of 56.5% of GPs could treat urethral discharge correctly as per the Namibian syndromic approach guidelines. None of the GPs could demonstrate the correct treatment of genital ulcer (whether they received medical aid or not) as recommended in the syndromic approach guidelines in Namibia (GRN, 1999; 2000). Only 28% of the GPs could demonstrate the correct treatment of Pelvic Inflammatory Disease (PID) as per the syndromic management of the STIs. For patients without medical aid the drugs prescribed and their dosages for PID are correct but the frequencies are not in line with the guidelines as for patients with medical aid.

**Discussion:**

In general, patients presenting with STIs to the GPs in private practices are not given quality of care because not all private general practitioners have time to do investigations, counseling, give condoms and to notify the partners of those with urethral discharge, genital ulcers and PID looking for treatment.

## 1. Introduction

### 1.1 Background

Common sexually transmitted infections can have severe consequences for individuals and communities all over the world and need to be addressed in an appropriate way within a specific framework of treatment (United Nations Fund for Population Activities 2004). In many parts of Africa, private general practitioners (PGPs) provide a significant proportion of primary-level health care services. One of the important conditions for which patients seek care in the private sector is sexually transmitted infections (STIs). STIs are epidemic in most parts of the world and provide a unique set of challenges for the nurse and physician. Even countries with relatively accessible public sector health facilities that provide STI free at the point of delivery have more than 50% of all their STI patients preferring to be seen by PGPs because of greater levels of privacy and a perception that the quality of care is better in the private sector than in the public sector (Chabikuli, Schneider, & Brugha, 2004; Smeltzer & Bare, 2000).

According to literature a large number of STI cases in the private sector are treated ineffectively and it is therefore considered essential to create awareness amongst the community regarding the management of STIs (United Nations Fund for Population Activities, 2004). The current situation of unregulated ‘therapeutic chaos’ in the private sector also has an equity dimension, as the poor tend to use informal and unqualified providers and are thus victim to unscrupulous practices. The lack of an effective and appropriate accountability framework for the private sector, coupled with the information asymmetry that does not enable patients to put pressure on providers, makes it difficult to ensure evidence-based practice and ethical behaviour in the private sector (Mayaud & Mabey, 2004; South African Health Review, 1999; United Nations Fund for Population Activities, 2004).

STIs, including the human immunodeficiency virus (HIV), are the most common cause of illness in Namibia, posing social and economic consequences. In 1998, STIs were ranked eighth amongst causes of outpatient consultation, with 80 000 reported consultations (Government of the Republic of Namibia, 1999 & 2000). The presence of HIV has changed and increased the importance of STI control, due to the strong correlation between the spread of conventional STIs and HIV transmission. Ulcerative STIs continue to facilitate high transmission of HIV because of the broken skin continuity making an easy port of entry for the HI virus and other infections. The presence of HIV has further affected the aetiology of STIs, causing the emergence of viral STIs such as herpes simplex to increase due to immune suppression. Antimicrobial resistance of several STI pathogens continues to increase over time, making treatment failure a common phenomenon in most STI-control programmes. Infection with one STI suggests the possibility of infection with other organisms as well (Smeltzer & Bare, 2000; United Nations Fund for Population Activities, 2004).

The complications caused by failure to diagnose and treat STIs include infertility, congenital abnormality, adverse neurological condition, cardiovascular risks, ectopic pregnancy, anogenital cancer and even premature death of neonates to mention but a few.

Therefore, control of STIs in a country with a heavy burden of HIV like Namibia, remains a public health emergency needing adequate funding and support. Political will to prioritise interventions pertaining to STI control remains the central thrust to effective control. Furthermore, control of HIV that leaves out strengthening of the STI control programme is bound to fail because, while HIV programmes continue to reach more people, emerging ulcerative STIs continue to facilitate further transmission of HIV.

Namibia has experienced a heavy STI burden that ranks 8th among all hospital consultations. Syndromic management was introduced in 1995 as an intervention for STI control. However, due to lack of human resources and capacity, implementation is not smooth as anticipated. A crucial aspect of STI control; training and supervision of all health workers rendering STI services was and is still weak. After 2001 an attempt was made to strengthen this aspect. This required consistent financial assistance. Strengthening STI training was done as from 2003, however clear data on the exact number of people trained is lacking. Supervision and ongoing training was not well monitored and coordinated in general. However, Namibia has national guidelines on the syndromic management of STIs at all health facilities and it is expected that health workers apply these guidelines in their dealings with STI patients.

### 1.2 Definition of Concepts

#### 1.2.1 Sexually Transmitted Infections (STIs)

An infection that can be transferred, from one person to another, through sexual contact. In this context, sexual contact is more than just sexual intercourse (vaginal and anal) and also includes kissing, oral-genital contact, and the use of sexual ‘toys’ such as vibrators (*Webster’s New World Medical Dictionary*, 2008). It is generally defined as any disease that can spread through sexual intercourse (Hornby, 2005).

#### 1.2.2 General Practitioner (GP)

A physician whose practice consists of providing ongoing care, covering a variety of medical problems in patients of all ages, often including referral to appropriate specialists. Also called the *family doctor* (*The American Heritage Stedman’s Medical Dictionary* 1995), the GP is furthermore defined as a doctor who is trained in general medicine and who treats patients in a local community rather than in a hospital (Hornby, 2005). A private general practitioner (PGP) links to the above definition referring to a general practitioner who owns a private practice

#### 1.2.3 State Health Services

In Namibia, where the study was conducted, this refers to health service rendered by any state health facility.

### 2. Objectives

The main objective for this study was: to describe the quality of STI treatment and care by PGPs in Windhoek in relation to national policy and treatment guidelines.

The specific objectives were to:

- assess the delivery of STI treatment by the private sector with reference to the national recommended guidelines for the syndromic management of STIs in Windhoek
- determine the availability of resources and items in the consulting rooms of the PGP in that are important for STI treatment
- describe recommendations for the improvement of STI treatment provided by PGPs.

## 3. Research Method and Design

This was a cross sectional quantitative study which aimed at assessing the quality of STI management by the private general practitioners in Windhoek (Burns & Grove, 2005). Quantitative variables were measured, analyzed and compared in among the various private general practitioners in relation to specific treatment of various STIs as per STI guidelines. This was done by talking to the service providers and in comparison to what is stipulated in the policy and STI Syndromic Treatment Guidelines (MoHSS, 1999).

### 3.1 Study Population, Sampling and Sample Size

The study population included all the PGPs in Windhoek, STI policy and treatment guidelines for STIs Syndromic treatment in Namibia.

Convenient sampling was applied by identifying the private general practitioners in the telephone directory, telephoned them and requested if they would participate in the study. Face to face interview was conducted with each GP who agreed to be part of the study. A total sample of50 PGPs took part in the study. This was a pilot study among the PGPs in Windhoek to inform a lager study in SADC.

### 3.2 Data-Collection Methods

Various methods of data collection were used in this study.

Firstly, a review of available country policy documents on STI management and surveillance, as well as the policy with regard to private primary care providers, using a checklist, was conducted. The review of policy documents was necessary to assess what the guidelines stipulate in terms of STI treatment and to determine whether PGPs follow these national guidelines in their treatment of STIs. Face to face interviews were conducted with all the respondents who were willing to participate in the study, using a structured questionnaire. Furthermore, an observation was done using the checklist to identify the type of resources and items that are in the consulting room that is important in the treatment of STIs in Namibia.

### 3.3 Data Analysis

With the assistance of a statistician data were analysed using the Microsoft Excel software. Frequency table and figureswere used to present the results in a meaningful way.

### 3.4 Ethical Considerations

The following ethical aspects were observed and considered:

- Written permission was obtained from the Ministry of Health and Social Services to conduct this study and a letter of ethical clearance was issued in this regard.
- Informed consent (verbal) was obtained from all the individual participants in the study.
- Participation was voluntary and participants had the freedom to withdraw from the interview or focus-group discussions at any stage.
- Confidentiality and anonymity was observed during all phases of data collection and reporting (Burns & Grove 2005).

## 4. The Main Findings

## Questionnaire Outcomes

Below are the findings from the structured interviews conducted with private general practitioners in Windhoek who participated in this study.

### 4.1 Year of Qualification

The private general practitioners interviewed were fifty in total and qualified between 1969 and 2004 as displayed in the Table 1 below.

**Table 1:** Year of qualification

Year of qualification	Number of doctors	Percentages
1969	1	2%
1970 – 1979	7	14%
1980 – 1989	20	40%
1990 – 1999	14	28%
2000 – 2005	8	16%
Total	50	100%

The majority of private general practitioners qualified between 1980 and 1989. The mean (average) year of qualification is 1989, implying that many of the doctors qualified in the year 1989. The first doctor qualified in 1969, and the latest doctor qualified in 2004.

It was very surprising to note that a person with a one year medical experience (qualified in 2004) is allowed to practice private practice in Namibia and for this matter practicing alone. This automatically calls for compromising the quality of care provided in general.

### 4.2 Post Graduate Medical Qualification

The study looked at post-basic medical qualifications as displayed in Table 2.

**Table 2:** General information on general practitioners and their practices

General information on general practitioner and practice	Response	
Post graduate Qualification	Yes	54%
	No	46%

Status of Medical practice	Alone	54%
	Group	46%

Sector of Employment	Private	90%
	Public	10%

Dispense Medication at Practice	Yes	70%
	No	30%

Practice Days Open	5 days	62%
	6 days	24%
	7 days	14%

As indicated in Table 2, 54% (n=27) of the private general practitioners have obtained a post graduate qualification. The majority has done a diploma in anesthetic, a few has master in public health and or a diploma in management. None of the private general practitioners interviewed indicated specific training in the syndromic management of the STIs as the focus of their studies at all.

### 4.3 Status of Medical Practice

The study also wanted to ascertain if the PGPs practice alone and or in groups and the results are indicated in Table 2.

The majority 54% (n=27) of private general practitioners work alone rather than in group practice. It was during this section when some private general practitioners expressed their concerns that private general practitioners in Namibia are left alone without having a watchful eye controlling their practices. Some stated that it is hard to know what your fellow colleagues are doing even if one wonders what quality of services they render when receiving patients from then.

### 4.4 Sector of Employment

The study also wanted to determine if some of the PGPs work in both public and private sector and the results was shocking, 90% (n=45) of the private general practitioners are purely in private practice and only 10% (n=5) of them practice also in the public health sector.

### 4.5 Dispensing of Medication

From 50 private general practitioners interviewed, only 30% (n=15) do not dispense medicine in their practices, while 70% (n=35) do dispense medicine to their patients (Table 2).

### 4.6 Patients on Medical Aid

An average of 86% of all patients seen by private general practitioners is on a medical aid. This variable confirms the sentiment that to have or not have a medical aid is a determinant of choosing to be a private or a state patient in Namibia.

### 4.7 Practice Days per Week

Practices of private general practitioners interviewed were open for an average of at least 6 days per week which increase the accessibility and availability of STI services for those using the services of the private general practitioners in Windhoek.

The average number of days most practices are open is 6. However, the majority 62% of the doctors’ practices are open for 5 days a week, while 24% are open for six (6) days, with only 14% open for seven (7) days. This range of days for service delivery ensures the accessibility of STIs services to the patients across the week as offered by the general private general practitioners.

### 4.8 Patients Seen a Day Prior to the Interview, and the Portion of STI Patients

An average of 23 patients was seen in general by the practitioner the day prior to the interview. All the private general practitioners indicate that they see patients with STI in their practice which implies that STIs patients are not restricted to public health facilities alone.

On average, each general practitioner examined one patient with STI a day before the interview. The highest STI cases per day seen by a general practitioner are five (5), however, there are some GPs with zero cases of STI patients seen a day prior to the interview and usually this is determined by the area where the practice is situated and the type of patients they see. One GP indicated that *“all my patients are from the upper class who knows how to take care of them and are unlikely to contract any STI”*.

### 4.9 Continuous Medical Education Sessions Attended

It was evident that all private general practitioners are aware of the importance of continuous education sessions that were offered during the last six months. Attendance of such continuous medical education did not show any particular pattern as can be seen below.

**Table 3:** Continuous medical education sessions attended

Number of CME sessions	Frequency	Cumulative %
0	4	8.33%
6	15	39.58%
11	19	79.17%
17	6	91.67%
23	2	95.83%
29	0	95.83%
34	1	97.92%
More	1	100.00%

**Figure 1:**
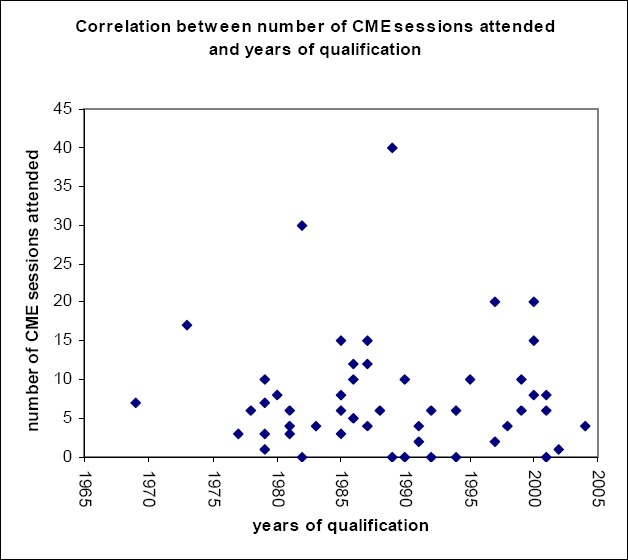
Correlation between number of CME sessions attended and years of qualification

The statistics do not show any correlation between years of qualification and the number of CME sessions attended. Almost all the private general practitioners interviewed have attended some sort of CME sessions except four of them. However, we observed that 92% (n= 46) of the GPs surveyed attended less than 20 sessions. The GP with the highest number of CME sessions (40) qualified in 1989. Each GP is supposed to have 60 CME sessions in one year. Each GP is supposed to have 60 CME Units in one year.

### 4.10 Management of Male Patients on Medical Aid Presenting with Urethral Discharge for the First Time

The study wanted to assess how the GPs treat specific STIs in general and whether they follow any guideline in their treatment. The result below represents how they treat urethral discharge.

For a male patient on medical aid with urethral discharge, most doctors prescribe, amongst other drugs, Doxcycline (56%) and Ciprobay (57%), Flagyl (18%) and Rocerphine (14%). The other commonly used drugs are Zithromax (16%), and Tetracycline (3%). An average of 56.5% of GPs treats urethral discharge correctly as per the syndromic approach. The rest give more than two drugs which is not recommended.

**Table 4:** Drug Treatment of Urethral discharge for patient with and without medical aid

Drug	With medical aid	Without medical aid
Doxcycline	56%	60%
Ciprobay	57%	54%
Flagyl	18%	24%
Rocerphine	14%	26%
Zithromax (Azithromycin)	16%	12%
Others		32%

For male patient without medical aid presenting with urethral discharge, most doctors prescribe amongst others Doxcycline (60%) and Ciprobay (54%), Flagyl (24%) and Rocerphine (26%). The other commonly used drugs are Zithromax (12%), and others (32%). An average of 57% (n=28) of the GPs treat urethral discharge correctly as recommended in the Syndromic Approach and the rest add other drugs as treatment.

Whether one has medical aid or not, the drug of choice remain Doxcycline and Cyprobay for the private general practitioners as one can see in figures 10 & 11. However, about 43% of the GPs prescribe drugs that are not recommended as treatment for urethral discharge to patients.

Comparing the treatment of male patients with urethral discharge between patients with & without medical aid, it can be observed that Doxcycline and Cyprobay are commonly prescribed in line with the syndromic approach.

There are few medications which are not prescribed to patients without medical aid. These are Zithromax, Augmentine, Erythromycin and Tarivid.

When managing a male patient on medical aid with urethral discharge for the first time, the majority of doctors 33% (n=30) do investigations, while 32% (n=29) of them or provide health education/counseling, 26% (n=24) doctors prompt partner notification and only 9% (n=8) doctors give condoms. From observation, no condoms are available in consulting rooms despite that the Ministry of Health and Social Services provide male condoms free of charge in Namibia.

### 4.11 Management of Male Patients without Medical Aid Presenting with Urethral Discharge First Time

The study wanted to determine how they manage a male patient with urethral discharge with or without medical aid for the first time and the findings was as follow:

People without medical aid are given more counseling and more investigations, followed by partner notification as indicated in Table 5.

**Table 5:** Management of male patients on with and without medical aid presenting with urethral discharge first time

Management	With medical aid	Without medical aid
Partner notification	26%	26%
Give condoms	9%	8%
Investigations	33%	31%
Health counseling	32%	35%

Comparing the management of first time male patients presenting with urethral discharge with & without medical aid, the management pattern looks similar, with investigations and counseling done by many doctors more than the partner notification and condom distribution.

### 4.12 Management of Patients on Medical Aid Presenting with Genital Ulcer for the First Time

The researchers wanted to determine how the private general practitioners treat a patient presenting with genital ulcer and the responses are as displayed and explained below in Table 6.

**Table 6:** Drug treatment of genital ulcer for patient with and without medical aid

Drug treatment	With medical aid	Without medical aid
Doxcycline	26%	24%
Ciprobay	14%	18%
Penicillin	14%	12%
Rocerphine	10%	10%
Erythromycin	15%	15%
Zelitrex	12%	10%

All GPs interviewed treat genital ulcer wrongly. In the treatment of patients without medical aid with genital ulcer, Doxycyline plus Rocephine and Benzathine Penicillin are commonly prescribed by the private general practitioners, followed by Ciprobay and Erithromycine. Other drugs such as a combination of Ciprobay, Erythromycine and Rocephine are prescribed only by some doctors and are not common among all the doctors. For the patients with medical aid, Doxcycline and Rocephine were mostly prescribed.

Whether patients have or do not have medical aid, the treatment was in most cases wrong and or incomplete when they are treated for the genital ulcers.

From the table below 72% (n=36) of the private general practitioners do investigations, however only 14% (n=7) of them provide condoms to their patients and 40% (n=20) notify the partner verbally. There was no notification slips for partner notification in any of the practice visited at all.

From the table below 48% (n=24) of the private general practitioners do investigations, however only 26% (n=13) of them provide condoms to their patients and 36 % (n=18) notify the partner verbally.

**Table 7:** Comparison of non-drug management of patients presenting with genital ulcer for the first time

Activity	With medical aid	Without medical aid
Investigations	72%	48%
Health education/counseling	46%	50%
Give condoms	14%	26%
Partner notification	40%	36%

In general it appears that when you have a medical aid, you are likely to be investigated compared to those patients without a medical aid.

### 4.13 Management of Female Patients with Medical Aid Presenting with Pelvic Inflammatory Disease for the First Time

In the treatment of female patients with medical aid presenting with Pelvic Inflammatory Disease (PID), Flagyl 400mg bd for seven days (66%) and Ciprobay 500mg stat and or bd for five days (44%) are commonly prescribed by private general private general practitioners, followed by Doxycycline 100mg for seven days (38%) and Augmentin (10%). Other drugs are prescribed only by some doctors and not common among all the doctors.

What is clear is that only 28 % (n=14) of the GPs could demonstrate correct treatment of PID as recommended in the syndromic management of the STIs, where they gave the correct drug, correct dosage and frequency. The rest who had a correct drugs and dosage in most cases the frequency is not in line with what is recommended. What came out is that 72% (n=36) of the GPs did not treat PID correctly for patients with medical aid.

**Table 8:** Management of female patients with medical aid presenting with pelvic inflammatory disease for the first time

Activity	Frequency	Percentage
Investigations	26	52%
Health education/counseling	24	48%
Give condoms	5	10%
Partner notification	18	32%

From the table above 52% (n=26) of the private general practitioners do investigations, however only 10% (n=5) of them provide condoms to their patients and 32 % (n=18) notify the partner verbally.

### 4.14 Management of Female Patients without Medical Aid Presenting with Pelvic Inflammatory Disease for the First Time

In the treatment of female patients without medical aid presenting with Pelvic Inflammatory Disease (PID), Flagyl (58%) and Ciprobay (40%) are commonly prescribed by doctors, followed by Doxycycline (38%) and Augmentin (8%). Other drugs are prescribed only by some doctors and not common among all the doctors such as Penicilline, Vaginal Creams and Ampicillin to mention but a few.

In most cases the drugs and dosage for the drug prescribed as treatment for PID are correct but the frequencies are not in line with what is recommended just as for patients with medical aid.

**Figure 2:**
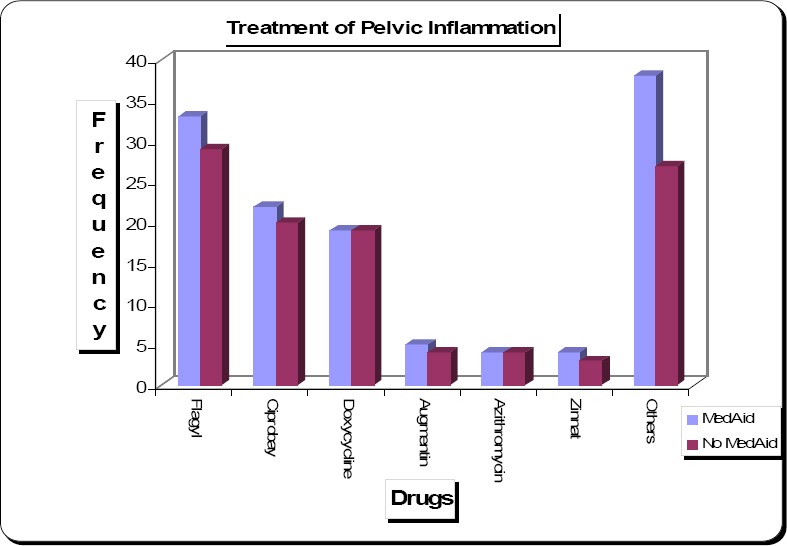
Treatment of Pelvic Infection Drugs like Flagyl, Ciprobay and Doxycycline remain the most favourite choice of treatment for both patients with and without medical aid.

**Table 9:** Management of female patients without medical aid presenting with pelvic inflammatory disease for the first time

Activity	Frequency	Percentage
Investigations	21	42%
Health education/counseling	17	34%
Give condoms	5	10%
Partner notification	12	24%

From the table above only 42% (n=21) of the private general practitioners per form investigations and only 10% (n=5) of them provide condoms to their patients and 24% (n=12) notify the partner verbally as they do not possess notification slips.

**Figure 3:**
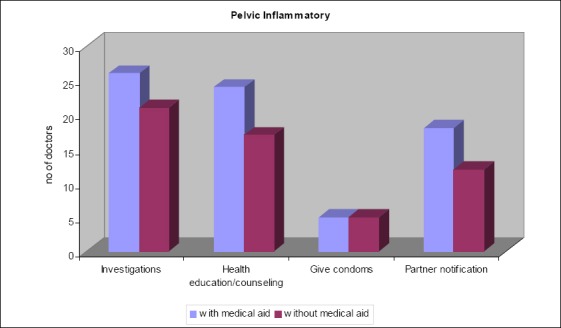
Comparison of management of female patients with and without medical aid presenting with pelvic inflammatory disease for the first time

Most doctors perform investigations 13% (n=26), counseling 48% (n=24) and partner notification 36% (n=18) on patients with medical aid than on those without medical aid. Giving of condoms appears to be performed without discrimination between those with or without medical aid.

Again this is not complying with what is recommended in the syndromic protocol as treatment for PID.

### 4.15 Items Available in Doctor’s Rooms

**Figure 4:**
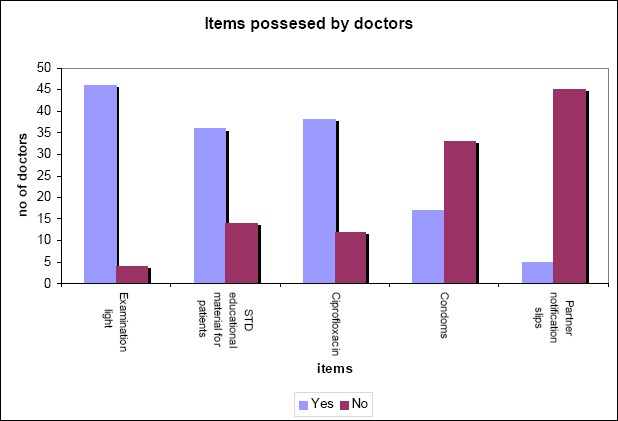
Items possessed by doctors

The research shows that 98% of the doctors under research had examination lights, 72% (n=36) have STD educational materials available and 76% (n=38) have Ciprofloxacin in their consulting rooms. On the contrary, 66% (n=33) of the doctors do not have condoms in their rooms, and 90% (n=45) of them do not possess partner notification slips.

### 4.16 Number of Vaginal Speculums Available in the Doctors’ Room

On average 13 speculums were available in the doctors consulting room and interestingly the private general practitioners mostly make use of disposable speculums.

### 4.17 Specific Protocol in Management of STI Patient

68% (n=34) of the private general practitioners reported that they follow specific STI protocols in managing STI patients, while 32 % (n=16) do not follow any specific protocol in management of STI patients.

**Figure 5:**
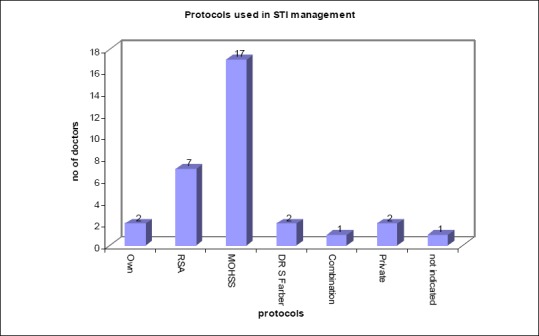
Specific protocol used in the management of STI

Of those private general practitioners who follow STI protocols in the treatment of STIs, they were asked to specify which protocol they use. Their responses are displayed in Figure 5.

Of the 50 doctors surveyed, 68% (n=34) used specific protocols in managing STI. Of those doctors using the STI protocols, the majority34% (n=17) use the MOHSS protocols. 14% (n=7) doctors use RSA protocols, and the remaining 16% (n=8) doctors either use their own, private protocol, Dr S Farber’s, a combination of protocols or did not indicate.

### 4.18 Availability of Mohss Protocol in Practice

Only 28% (n=14)of the private general practitioners had a copy of the Ministry of Health and Social Services´ protocol on management of STI in their practice while the majority 72% (n=36) of the private general practitioners do not have a copy of the MoHSS protocol on the management of STI in their practice.

### 4.19 Application of Syndromic Management of STI

The researchers wanted to determine if GPs do apply any specific syndromic protocol in the management of STIs and the responses are as follow.86% of the doctors follow syndromic management in treating STI patients. The 14% do not.

### 4.20 Tests Done on Women for Ante Natal Care

**Figure 6:**
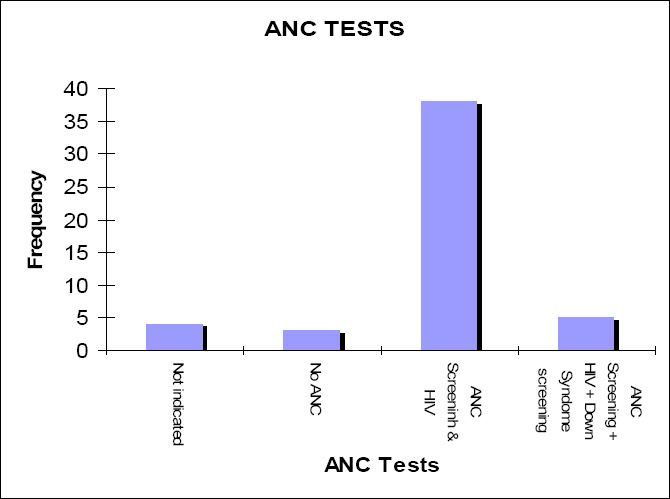
Testsdone on women for ante natal care Only three private general practitioners do not do any tests on women who come for ANC care.

## 5. Discussion of the Findings

As the findings indicate, amongst the PGPs interviewed, only 44% qualified after 1990. This implies that most of the PGPs in Windhoek are experienced practitioners who have been long enough in the medical field to ensure quality care provision to their patients. However, it was also shocking to find a person who just qualified in 2005 his internship practicing private practice alone. One would expect such a person to work in a group practice to enable oneself to gain practice from others. With this finding, the researchers questioned the process of ranting approval for private practice by the Ministry of Health and Social Services in Namibia. There seems not to be any requirement as long as you are a registered medical officer with the Medical and Dental Council.

Working in a group practice could help ensure quality care provision, as the private general practitioners could consult each other in case of doubt. However, the current state of affairs where the majority (54%) of the private general private general practitioners is mostly working alone could really raise concerns as expressed by some private general practitioners about the competencies of some of their colleagues in treating the STIs.

Some PGPS expressed their concerns that private practitioners in Namibia are left to themselves without having a watchful eye controlling their practices. Some stated that it is hard to know what your fellow colleagues are doing wondering what quality of services they render when receiving patients from their practices.

For a country with a critical shortage of medical doctors, one would expect the state to request the service of the private general private general practitioners to help alleviate the shortage for some hours a day in public health facilities. However, the private general private general practitioners who also work in the public sector (10%) indicated that the money paid for the service they render, is apparently not worth the sacrifices.

Continuous Medical Education is required from each professional to attend as stipulated in the Medical and Dental Act No of 2004. This is so important to ensure that private general practitioners are up to date with new development in their professions. However, it was observed that 92% (n=46) of the PGPs surveyed attended less than 20 sessions by the time of the study. The PGP with the highest CME sessions (40 sessions) was the oldest among them who qualified in 1989. This was encouraging to note that a person who have been long in the play field continued to update his knowledge and skills which should be exemplary to the young professionals.

The study revealed that only 56.6% of the PGPs treat urethral discharge correctly as recommended in the guidelines while the rest treat it contrary to the guidelines. This is of great concern because it compromises the quality of management of STI in Namibia. Following the syndromic approach in the management of STI was found to be cost effective in general and if all GPs could use the flow chart as recommended would improve the management of Urethral discharge as an STI (Mayaud & Mabey, 2004).

It is worth noting that private general practitioners prescribe different dosages for different duration for the same drugs indicated in the figures above irrespective whether you have medical aid or not. However, it is obvious that all private general practitioners have used 100mg for Doxcycline for seven days as recommended in the syndromic approach but when it comes to Ciprobay, the dosages varies from 500mg stat to 500mg for seven days and or 1gram twice a day for seven days. What was clear is that the dosages for Ciprobay are too varied and confusing and this need serious attention as patients need to be treated correctly and effectively. There is a danger of drug resistance and the enforcement of developing chronic conditions among patients seen by the private general practitioners (GRN, 1999; GRN, 2000).

In general, patient are not given quality care because not all private general practitioners have time to do investigations, counseling, give condoms and to notify the partners of those with urethral discharge looking for treatment. Education/counseling, notifying the partners of patients and giving of condoms to patients form a major part of the treatment regime of urethral discharge as per the syndromic protocol (GRN, 1999; Mayaud & Mabey, 2004). In such practices the GPs are running a risk of exposing their patients to re-infections and this also put the patient at risk of contracting HIV in the long run as they are not informed of the danger involved if both partners do not get treatment.

Literature recommend that to treat and control STI effectively, the following strategies should be part of the treatment such as education and counseling of the person at risk on the ways to avoid STI infection through changes in sexual behaviors and use of recommended prevention services; identification of asymptomatically infected persons and of symptomatic persons unlikely to seek diagnostic and treatment services; effective diagnosis, treatment, and counseling of infected persons; and evaluation, treatment and counseling of sex partner of persons who are infected with an STI (GRN, 1999; Kimberly & Workowski, 2010). It was clear that genital ulcer is wrongly managed among the PGPs and this worrying and needs speedy action to protect the public from the risk of developing drug resistance in general. The syndromic approach recommends Ceftriaxone 250mg intramuscular injection stat plus Benzathine Penicilline 2.4 million units intramuscular as the first line drug of treatment for Genital Ulcer in Namibia (GRN, 1999).

Furthermore, the results revealed that poor quality management of patients with genital ulcer, and this calls for urgent action from the Ministry of health and social services to correct this practice in terms of training to update the PGP in the correct treatment of STI in general and demand that they use the guidelines as prescribed. Some PGP actually indicated they are not familiar with the concept of “syndromic approach” and called on Government to orient them about this treatment approach. Its seems there is no rule/regulations requiring all medical practitioners to prove that they are up to date with new treatment guidelines in general for any type health conditions in Namibia.

It was clear that genital ulcer is wrongly treated among most private general practitioners and this is worrying and need action to protect the public. The syndromic approach recommends Ceftriaxone 250mg intramuscular injection (imi) stat and Benzathine Penicilline 2.4 million units imi as the first line drug of treatment for genital ulcer (GRN, 1999 & 2000). The results revealed that poor quality management of patients with genital ulcers, because patients are wrongly treated and steps need to be undertaken to correct the situation in terms of training to update the private general practitioners in the genital ulcer treatment. Some GPs indicated that they are not familiar with the concept of “syndromic management” and the Ministry needs to take up the issue to orient the GPs around the syndromic management of the STI in Namibia.

It is stated in the literature that STI case management using the syndromic approach is feasible, adaptable, and cost effective. It works best for the management of genital ulcer disease and urethral discharge in men (Mayaud & Mabey, 2004). If only the public sector could involve the GPs when introducing new treatment strategies and enforce its use, the change might not be feasible among the GPs.

It is worth noting that private general practitioners prescribe different dosages for different duration for the same drug for the drugs indicated in the figures above. It was noted also that the dosages for Penicillin, Ceftraixone and Flagyl were consistent among most private general practitioners who prescribe them for this condition although the duration/frequencies differed a lot. In some cases Ceftriaxone was prescribed 250 mg stat three times which meant that a patient had to come to the practice for three days to receive this injection. The same goes for pencilline where it was prescribed 2.4 million units for seven days. This sounds outrageous but this is what the data revealed.

With regard to the non-drug treatment of patients presenting with genital ulcer, one would conclude that to have or not to have a medical aid does not has an impact in the type of treatment provided as seen from the graph above. Although most doctors indicated that they do investigations more for those with medical aid as oppose to those without medical aid. It is sad to note that giving condoms and notifying of partners is done least by the private general practitioners when treating genital ulcers exposing both partners to other sexually transmitted infections such as HIV. The results are similar to the findings in South Africa where most private doctors reported supplying condoms and several reported counseling patients but none of them provide partner treatment cares or written health education, indicating that comprehensive syndromic treatment was not being practiced at all (Wilkinson, Abdool, Karim, Lurie, & Harrison, 2001).

By implication this means that the private general practitioners do not allocate enough time to discuss conditions with their patients so that the patients can be empowered to protect themselves from becoming re-infected. The other reason might be that the GPs would like to see such patients keep coming back with the same infections and serve as their source of income, which could bring in the ethical and moral issues in the practice. The whole scenario could indicate the ignorance among the private general practitioners with regard to the treatment of any STI in general. Health education/Counseling and condom giving is one of the treatment of any STI worldwide (GRN, 1999; Wilkinson et al., 2001).

However, there could be other reasons why the PGPs are not treating STI patients effectively which could include individual doctors be in competition with each other as there is little opportunities to bring them together to share experience and to develop solutions to problems; little history of public-private sector interactions other than passive referral of case from private practitioners to public hospitals; resistance to change due to a strong ethos of individual practitioner as decision-maker; economic reasons as the main force determining practice patterns and little interest in using STI syndromic packets and own drugs (Wilkinson et al., 2001).

It is worth noting that the education material available is mainly for the private general practitioners´ references and not for patients to read while waiting for their turn. Such educational materials are mostly written in English and Afrikaans meaning that patients, who cannot read any of the two languages, are excluded from the information. This could confirm the fact that there is no cooperation between the public and the private sectors when it comes to the management of STIs.

## 6. Significance of the Study

The findings can be used by the Ministry of health and Social Services to strengthen their relationship with the PGPs in general. It further describe how the PGPs manage STIs and based on that a strategy could be developed to empower the PGPs in this area either reinforce the availability of the guidelines in their private practices and initiate training for the PGPs in general. The statutory Medical and Dental Council gets informed to strengthen their supervision for those in general practice in Namibia

## 7. Limitations of the Study

The study was just restricted to Windhoek and among the private practitioners only, therefore the results cannot be generalized. The practitioners were selected purposefully and conveniently which could introduce bias in the data.

## 8. Conclusions

The researcher came to a conclusion that the guidelines for the syndromic management for STI in Namibia do exist in Namibia but this is hardly known and or followed by the entire private general practitioners. Those private general practitioners, who follow the protocol, do so inconsistently.

Urethral Discharge is to a certain extend correctly managed by the PGPs but this cannot be said for Genital ulcers not for PID management. Different drugs are prescribed with different dosage, duration and frequencies which can facilitate drug resistance as well as development of chronic conditions in patients. Condoms are not offered from the consulting rooms and their counseling as well as investigations for their patients with STIs is non-existence.

## Recommendations

The researchers would like to recommend to the Ministry to consider all the recommendations given by the GPs and try to implement them for the sake of preserving quality care provide to the public such as acceptance of referrals from GPs, offer training to GPs for any new strategies introduced in treatment, distribute condoms and notification slips at all private practices in the country, regular visiting such practices etc.

There is need of interactions between public and private sector at various levels to ensure that curable STIs are appropriately managed and that national guideline for STI management is adhered to.

Awareness creation among the GPs with regard to the public health importance of STI needs to be raised for the GPs to participate in the STI control programme and ask them to have a representative on such forum.

Furthermore, the policy with regard to granting permission for one to practice privately need to be revised so that those going private should have enough experience and skills in working all alone.

The Medical and Dental Council should make its present in the country visible especially among the GPs to help ensure the good standard of care at all times by being a “living watchful eye”.

There is need for the Ministry to improve the care provided at their health facilities and ensure that the processes at health facilities can encourage patients to come back and not to turn them away because of the long queues experienced currently.

## References

[ref1] Burns N. B, Grove S. K (2005). The practice of nursing research: Conduct, critique and utilization.

[ref2] Chabikuli N, Schneider H, Brugha R (2004). Working with the private providers on the control of Sexually Transmitted Infections. A manual for district programme managers. The centre health policy and London School of Hygiene andTropical Medicine.

[ref3] Mayaud P, Mabey D (2004). Approaches to the control of sexually transmitted infections in developing countries: old problems and new challenges. Sex. Transm. Inf.

[ref4] (2004). Medical and Dental Act. Government of the Republic of Namibia.

[ref5] (1999). Government of Republic of Namibia. Guidelines for the syndromic management of sexually transmitted diseases.

[ref6] (2000). Government of Republic of Namibia. Primary Health Care Drug Formulary.

[ref7] (2004). Government of Republic of Namibia. The Technical Efficiency of District Hospitals in Namibia, July, 2004, MoHSS.

[ref8] Hornby S (2005). Oxford advanced learner’s dictionary of current English.

[ref9] Kimberly Workowsk, Bermen S (2010). Sexually Transmitted Diseases guidelines, National Center for HIV/AIDS, Viral heapatitis, STD and TB prevention.

[ref10] Smeltzer S. C, Bare B. G (2000). Textbook of medical-surgical nursing.

[ref11] (1999). South African Health Review. The briefing summary: patient choice of primary care and the need to influence quality of STD care in the private sector.

[ref12] (1995). The American Heritage Stedman’s Medical Dictionary.

[ref13] (2004). United Nations Fund for Population Activities (UNFPA). Sexually transmitted infections: Breaking the cycle of transmission UNFPA, New York.

[ref14] (2008). Webster’s New World Medical Dictionary.

[ref15] Wilkinson D, Karim S. S, Lurie M, Harrison A (2001). Public-Private Health Sector Partnership for STD Contorl in South African perspectives. From the Hlabisa Experiences. SAM June 20101.

